# Targeted RNA base editing for therapeutic: mechanisms and advances

**DOI:** 10.1016/j.pscia.2025.100089

**Published:** 2025-08-18

**Authors:** Weikai Yan, Xiaocheng Weng

**Affiliations:** College of Chemistry and Molecular Sciences, State Key Laboratory of Metabolism and Regulation in Complex Organisms, Department of Otorhinolaryngology-Head and Neck Surgery, Zhongnan Hospital of Wuhan University, Taikang Center for Life and Medical Sciences, Wuhan University, Wuhan, 430072, China

**Keywords:** RNA editing, Point mutation, CRISPR-Cas, RNA modification

## Abstract

RNA base editing, which enables RNA base modification through effector proteins guided by targeting systems, is a powerful technology to correct disease-associated point mutations. Although overshadowed by CRISPR-based genome editing, RNA editing has seen rapid development in recent years, with significant improvements in efficiency and precision. In this review, we summarize the core components of RNA base editing systems (RNA-targeting systems and effector proteins) and describe major RNA editing types, including A-to-I, C-to-U, A-to-m^6^A/m^6^A-to-A, and U-to-Ψ base editors, along with their research progress. In addition, we systematically summarize the delivery methods of the developed RNA editors and their initial exploration in treating diseases caused by nonsense mutations. Finally, combining the current development status of the RNA editing related field, we reflect on the problems encountered in the current development of the RNA editing field and offer our own insights on the future development direction.

**Table:** List of abbreviations

Abbreviation	Full name
CRISPR	clustered regularly interspaced short palindromic repeats
Cas	CRISPR-associated proteins
DSBs	double-strand breaks
RNP	ribonucleoprotein
ADAR	adenosine deaminases acting on RNA
RBPs	RNA-binding domains
PUF	Pumilio and FBF
snoRNA	small nucleolar RNA
BG	benzylguanine
MCP	MS2 coat protein
IFN	interferon
Ψ	pseudouridine
NLS	nuclear localization signal
PTC	premature termination codon
NMD	nonsense-mediated mRNA decay
SAM	S-adenosyl methionine
m^6^A	*N*^6^-methyladenine
PAM	protospacer adjacent motif
hESCs	human embryonic stem cells
AAV	adeno-associated virus
LNP	lipid nanoparticle
HSCT	hematopoietic stem cell transplantation
ERT	enzyme replacement therapy
CF	cystic fibrosis
PBDs	peroxisome biogenesis disorders
DMD	Duchenne muscular dystrophy

## 1 Introduction

Clustered regularly interspaced short palindromic repeats (CRISPR)-CRISPR-associated protein 9 (Cas9) has been used to manipulate the genomes of cells since 2012 [1]. Such DNA editing technologies can alter genome sequences and induce mutations at amino acid sites, leading to phenotypic changes. In addition to double-strand breaks (DSBs) based DNA editing technologies, DNA base editors are promising tools for correcting point mutations at single-base resolution. Point mutations constitute the largest human pathogenic mutations (about 58 ​%). DNA base editors utilize effector proteins like deaminases and the Cas9 targeting system to precisely convert targeted base pairs [2]. However, permanent changes to the genome may lead to unwanted results, hindering their clinical application in therapeutics.

RNA base editing is a natural process with diverse roles in metazoans [3], one of its functions is to facilitate immune evasion by modifying endogenous RNA [4]. Like DNA base editors, artificial RNA base editors use effector proteins and targeting systems to achieve precise modification of a nucleotide. RNA editing, as an inherent life phenomenon within cells, is the method of additional modification of RNA single bases and should be called artificial RNA editing. However, researchers still directly use the term RNA editing to describe this artificial process. Unlike DNA editing, RNA base editing exhibits unique therapeutic advantages due to differences in target nucleotides. Specifically, RNA editing is reversible and doseable, as RNA degradation enables transient effects and natural error elimination. This transient nature enables RNA editing to serve as a safer therapeutic modality, circumventing ethical concerns associated with permanent germline alterations. Compared to other RNA therapeutics, such as small interfering RNA (siRNA), which require transcript knockdown and are unsuitable for all disease phenotypes [5], RNA base editing offers a unique advantage by modifying a single nucleotide.

**Fig. 1:**
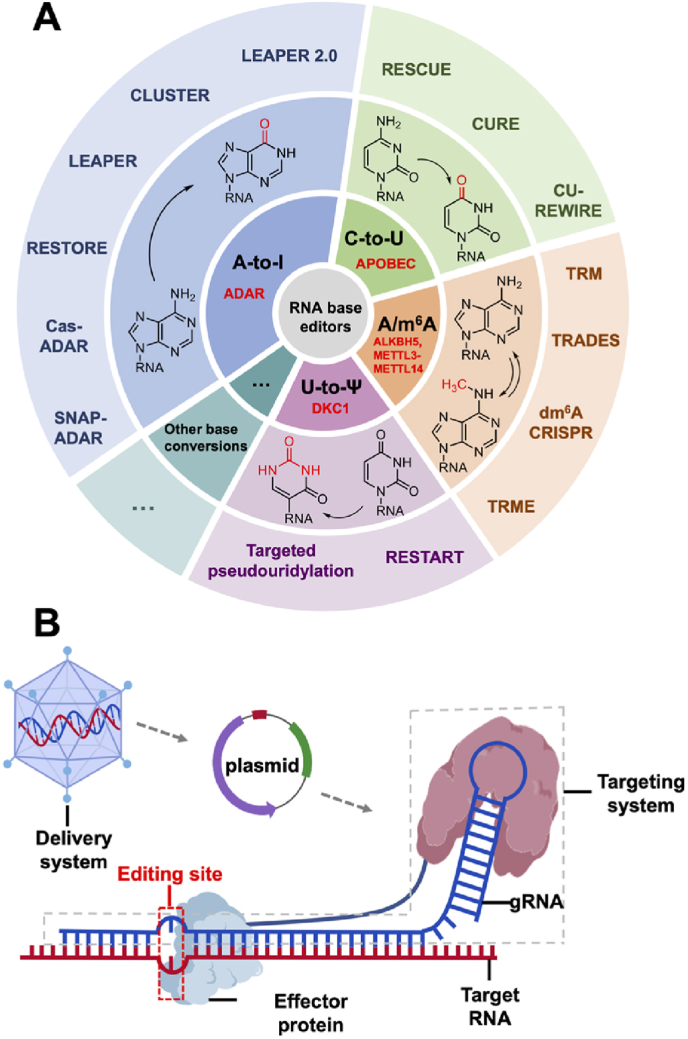
Overview of RNA base editors and elements of RNA base editing systems. **A**. A-to-I, C-to-U, A-to-m^6^A/m^6^A-to-A, and U-to-Ψ base editors are gradually developed recent years, but there exist other unexploited but potential base conversions for RNA base editing. **B.** A general RNA base editor mainly contains targeting system (e.g., Cas systems), effector protein (e.g., ADAR, exogenous or endogenous). Besides, delivery system is also vital for the medical application of RNA editors.

The key components of an artificial RNA base editor comprise two core elements: an RNA-targeting system and an effector protein. The targeting system recognizes specific RNA sequences to ensure precision, without which off-target editing may occur. Effector proteins are typically RNA-modifying enzymes (exogenous or endogenous) that mediate base conversions (e.g., C-to-U) or modifications (e.g., A-to-I). (Fig. 1A and B).

In this Review, we illustrate the key components of RNA base editors and summarize the existing programmable RNA base editors, including adenosine-to-inosine (A-to-I), cytidine-to-uridine (C-to-U), uridine-to-pseudouridine (U-to-Ψ), and adenosine-to-*N*^6^-methyladenosine (A-to-m^6^A)/m^6^A-to-A base editors (Fig. 1A). This article pays particular attention to RNA editing tools that convert standard bases into modified bases, which is also a booming but rarely introduced field at present. We also focus on the delivery methods of the RNA editor that has been developed, as this is crucial for its application in future treatments. Finally, we introduce some examples of RNA editing that have been preliminarily verified on cell models for therapeutic. Finally, in response to some of the current predicaments faced by RNA editors, such as off-target editing, we have also made corresponding summaries and, in combination with the development of related fields, predicted the possible future development directions of RNA editing.

## 2 Targeting systems of RNA base editors

**Fig. 2:**
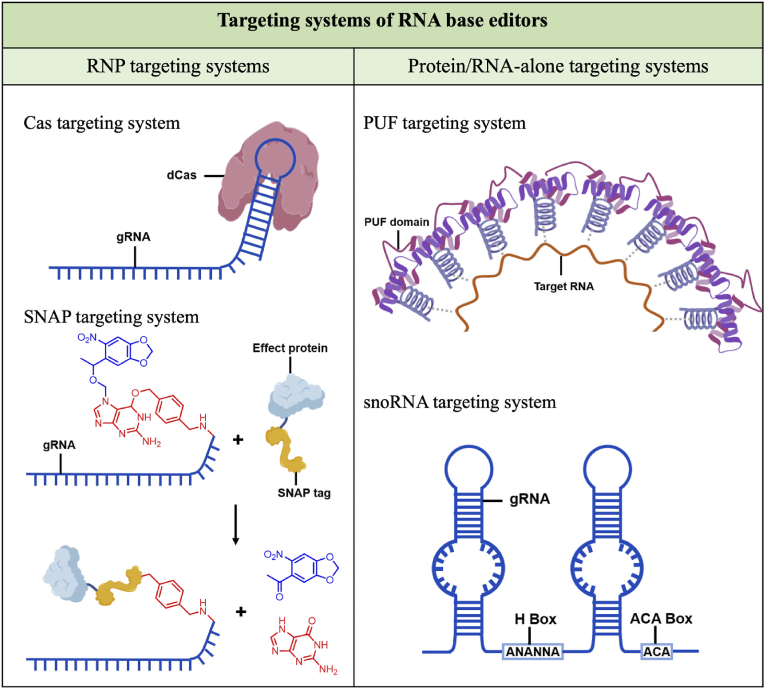
Targeting systems of RNA base editors: (1) RNP based targeting systems. Cas targeting system is the most used targeting system, SNAP-tag is a chemical tag for tying protein and RNA together. (2) Protein/RNA-alone targeting systems. PUF targeting system is a protein-alone targeting system, while (H/ACA box) snoRNA targeting system is RNA-alone.

**Fig. 3:**
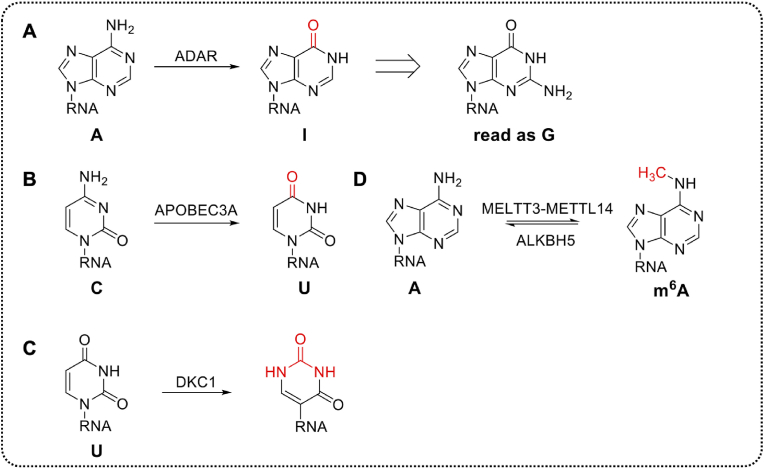
Base conversion, modification patterns and effector proteins of developed RNA base editors. **A.** A-to-I (read as G). **B.** C-to-U. **C.** U-to-Ψ. **D.** A-to-m^6^A/m^6^A-to-A.

Depending on the main substance acting, targeting systems of current RNA base editors can be divided into three types: (1) ribonucleoprotein (RNP) targeting systems (Fig. 2). (2) protein targeting systems. (3) RNA targeting systems (Fig. 2). Most effector proteins for RNA editing are RNA modification enzymes, which cause base conversion or base modification (Fig. 3).

### 2.1 RNP targeting systems

CRISPR-based systems are the most commonly used RNP targeting systems. Dead Cas proteins (dCas), namely inactivated Cas proteins, are devoid of nucleolytic activity, so it can be used to deliver functional cargo to targeting-sites without activating the digestion activity of Cas proteins [6] (Fig. 2). Based on the DNA-targeting CRISPR systems, RNA-targeting CRISPR systems were also been identified in recent years, the Cas13 system is the most representative among them. The Cas13 system belongs to the class 2 type VI systems [7], is an RNA-guided RNA endonuclease, and consists of two HEPN (higher eukaryotes and prokaryotes nucleotide-binding) domains. HEPN domains together form a composite RNase active center, which enables targeting RNA and then catalyzes RNA cleavage [8,9]. To generate dCas (dead Cas) protein, specific point mutations are introduced into the catalytic residues of the Cas nuclease domain. Inactivated dCas13 can only bind to target RNA, which makes it a remarkable targeting tool for RNA editing. Varieties of Cas13 proteins have been characterized and developed, including Cas13a [10], Cas13b [11], Cas13c [10], Cas13d [12], and Cas13X/Y [13], while their therapeutic applications are often limited by the delivering capacity of adeno-associated viruses (AAVs). Developing compact Cas13 proteins is also significant for the further medical application of RNA base editing. Yao et al. identified the Cas13j family, with LepCas13j (529aa) and ChiCas13j (424 aa), as the most compact Cas13 proteins. They used the miniaturized Cas13j proteins to construct compact A-to-I RNA base editors and highlight the potential of Cas13j to treat human diseases [14] (Table 1).

**Table 1:** The types and sizes of Cas proteins and effector proteins applied in RNA editing.

Protein/Ortholog		Size	Reference
Cas13a	LwaCas13a	1152 aa	[10]
Cas13b	PspCas13b	1092 aa	[11]
Cas13c	FpCas13c	1110 aa	[10]
Cas13d	RfxCas13d	967 aa	[12]
Cas13X(Cas13bt)	Cas13X.1	775 aa	[13]
Cas13Y(Cas13bt)	Cas13Y.1	790 aa	[13]
Cas13j	Lepcas13j	529 aa	[14]
	ChiCas13j	424 aa	[14]
ADAR	hADAR1	531 aa	[18]
	hADAR2	731 aa	[19]
APOBEC3A		199 aa	[20]
DKC1		515 aa	[21]
METTL3		580 aa	[22]
METTL14		456 aa	[22]
FTO		126 aa	[23]

In sgRNAs designed for various Cas proteins, a 30-bp spacer sequence is employed to ensure efficient target recognition through complementary base pairing with regions flanking the desired editing site. Furthermore, positioning the editing site centrally within this complementary region has been observed to yield higher editing efficiency.

Other RNP targeting systems are mainly based on the ligation or interaction of proteins with guide RNA (gRNA). SNAP-tag derives from the *O*^6^-benzylguanine DNA alkyltransferase, and it can be covalently linked to *O*^6^-benzylguanine. By modifying a 22-nt or 25-nt gRNA with benzylguanine, and labeling the effector protein with SNAP-tag, gRNA can have a covalent connection with SNAP-protein to form a RNA editing complex, so RNA editing events will occur on target RNA sites (the editing site is also centrally within the complementary region) [15] (Fig. 2). λN-peptide [16] and MS2-coat proteins [17] et al. have specific interactions with some special RNA secondary structures, by inducing the specific RNA sequence to gRNA and fusing λN-peptide or MS2-coat proteins to effector protein can also generate RNA base editors.

### 2.2 Protein-alone targeting systems and RNA-alone targeting systems

Most engineered RNA base editors require both protein and RNA to assemble RNA-protein complex; however, due to the existence of a large amount of endogenous proteins and RNAs, the assembly and co-folding of the RNA–protein complex can be the rate-limiting step that reduces editing efficiency and hinders its application [24]. Protein-alone or RNA-alone targeting systems may circumvent this problem.

Here presents a protein-alone targeting system, RNA binding scaffold of Pumilio and FBF homology (PUF) proteins contains eight repeat motifs, each of which can be reprogrammed to specifically bind any RNA base through interaction with the Watson–Crick edge [[25], [26], [27]] (Fig. 2). Reprogramming the PUF domain enables it to target almost any 8-nucleotide (8-nt) RNA sequences [56].

As mentioned above, delivery barriers hinder the therapeutic applications of many RNA editors, and exogenous effector proteins sometimes may cause immune responses. Using gRNA-alone targeting systems and recruiting endogenous effector proteins have been demonstrated to be feasible. Specially designed long linear or circular gRNA, chemically modified oligonucleotides, and small nucleolar RNA (snoRNA, Fig. 2), which contain both gRNA sequences with different length complementary regions and recruitment sequences of effector protein, have been applied in A-to-I editing and U-to-Ψ editing [28].

## 3 A-to-I base editors: from exogenous ADAR to endogenous ADAR

ADAR (adenosine deaminases acting on RNA) enzymes target adenosine bases in dsRNA, mediating the deamination of adenosine and producing inosines to introduce A-to-G mutations because inosines can form base pairing with cytosines and then biochemically interpreted as guanosines in RNA [29] (Fig. 3A). There are three ADAR proteins exist in human cells: ADAR1, ADAR2 and ADAR3 [30], but ADAR3 shows no editing activity. ADAR1 has two known isoforms: the longer isoform p150 and the N-terminally truncated isoform p110 [31]. All of those ADAR proteins contain dsRNA-binding domains (RBDs) and a C-terminal deaminase domain (DD), which guide the ADAR enzyme to substrates and convert adenosine to inosine through a base-flipping hydrolysis mechanism, respectively [32].

A-to-I editors, which exploit ADAR as the effector protein, are the pioneer of RNA base editors, and the most studied one. Both RNP targeting systems (SNAP-tag, λN-peptide, MS2-coat proteins, and CRISPR-based targeting system et al.) and protein/RNA-alone targeting systems (PUF domain or long RNA with a specific structure) have been used for targeting A-to-I editing sites. Over 20 versions of programmable A-to-I editors have been developed and used for therapeutics.

### 3.1 Exogenous ADARs for A-to-I editing

Earlier A-to-I editing systems mainly focused on tying targeting systems and effector proteins together, so that they can act simultaneously and in space. Exogenous ADARs seem to be an appropriate chance to realize site-specific A-to-I editing in vivo.

#### 3.1.1 SNAP-ADARs RNA base editors

**Fig. 4:**
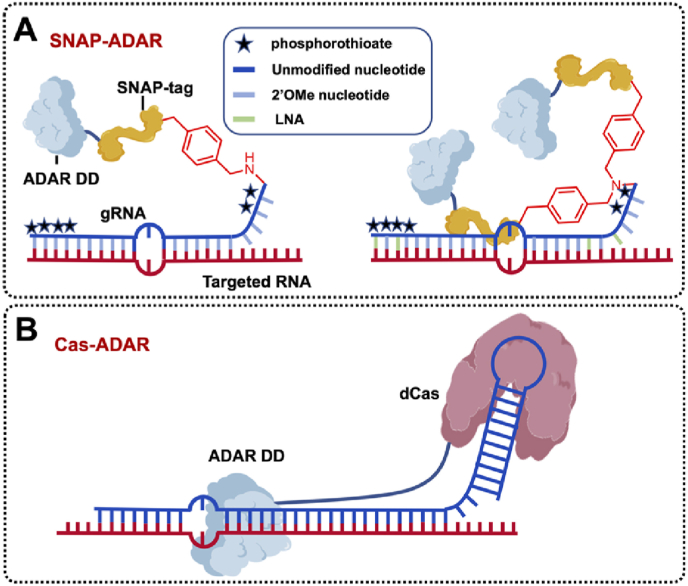
Exogenous ADAR for A-to-I RNA base editing. **A.** Diagram of SNAP-ADAR and improved SNAP-ADAR. A SNAP-tag was fused to ADAD DD, and it reacted with BG-modified gRNA, to form gRNA–deaminase conjugates. **B.** Diagram of Cas-ADAR. A dCas protein was fused to ADAR DD to direct the editing of targeted cytosine.

Thorsten Stafforst et al. developed a series of SNAP-ADAR tools for RNA A-to-I editing since 2012, and SNAP-ADAR is also considered the first artificial RNA base editors. Natural ADARs bind their substrates with N-terminally fused dsRNA-binding domains [33], so the priority among priorities of exploiting SNAP-ADAR is to direct ADAR enzymes for a specific reaction at a new substrate, that is to say, turn ADAR into a gRNA-dependent enzyme. They fused the isolated C-terminal deaminase domain (aa 798–1226) of hADAR1 to the C-terminal domain of a SNAP-tag, which is an engineered *O*^6^-alkylguanine-DNA-alkyl transferase, hence removing the original substrate-binding domain of hADAR1 successfully. Then the SNAP-tag reacted with 5′-*O*-benzylguanine (BG)-modified gRNA to form the covalent gRNA–deaminase conjugates [18] quantitatively within an hour (at a probe concentration of roughly 1 ​μM) [34](Fig. 4A). In 2014, inspired by chemically modified antisense oligomers, they demonstrated that chemically modified gRNA in the SNAP-ADAR tool is a reliable means to suppress overreaction and steer selectivity, and even larger chemical modification groups including global 2′-methoxy and terminal phosphothioate groups, can be adopted (with a three-nucleotides small gap). They also proved that the SNAP-ADAR tool is functional inside the cells [35]. Later, several measures have been put forward to optimize the SNAP-ADAR tool, such as light-induced self-assembly [36], genomic integration of the precise editase [37], extremely short gRNA [37], optimizing the secondary structure [38], and inducing mismatch base pairs near the target sites [38]. These optimized SNAP-ADAR tools realized up to 90 ​% relative efficiency on cell endogenous transcripts.

In 2024, they developed an improved SNAP-ADAR tool, which enables efficient RNA base editing to interfere with post-translational protein modification. Notably, locked nucleic acid (LNA) was induced in gRNA, and a bivalent linker for recruitment of two SNAP-ADAR proteins per guide RNA (BisBG) was also applied to improve the efficiency of A-to-I editing [39].

#### 3.1.2 λN-ADAR and MCP-ADAR

The λN-ADAR system, pioneered by Montiel-Gonzalez et al., utilizes a fusion protein combining the λ-phage N-peptide with the catalytic deaminase domain (DD) of human ADAR2. This fusion is recruited to target mRNAs via a synthetic guide RNA containing a complementary antisense sequence and a cognate box B RNA hairpin. This platform achieved up to 70% editing efficiency on reporter in HEK293T cells [40]. Building on this concept of engineered recruitment, the MCP-ADAR system replaces the λN peptide with the MS2 coat protein (MCP) fused to hyperactive ADAR DDs. Guide RNAs incorporate MS2 hairpins flanking the antisense domain, enabling robust recruitment. This system demonstrated high on-target editing efficiencies (up to ∼30%) across diverse endogenous transcripts in human cells and achieved functional correction in disease models [17].

λN-ADAR and MCP-ADAR platforms face significant challenges. Specificity remains a primary concern: both editing tools showed notable off-target editing, cytotoxicity, and sequence preference. These limitations highlight the need for further engineering to enhance specificity, reduce toxicity, and optimize delivery strategies for robust and safe therapeutic RNA editing.

#### 3.1.3 Cas-ADARs RNA base editors

Feng Zhang et al., pioneers of CRISPR-Cas9 genome editing, first harnessed CRISPR-Cas tools into RNA base editing (Cas-ADARs) in 2017. They identified a Cas13b ortholog from *Prevotella* sp. *P5-125* (PspCas13b), then fused the ADAR2 DD with the E488Q mutation to catalytically inactive PspCas13b, they called it REPAIRv1 (Fig. 4B). As the first dCas-guided RNA base editor, the editing efficiency of REPAIRv1 just obtained 28 ​% in HEK293T cells with the massive bystander editing and global off-target editing. Using a rational mutagenesis scheme, they generated a more precise variant called REPAIRv2, which achieved more than 919-fold higher specificity [41]. However, the therapeutic application of REPAIRv1 and REPAIRv2 is still limited by their size, far exceeding the packaging capacity of AAVs. Later, they identified ultrasmall Cas13 proteins: Cas13bt1 and Cas13bt3. Besides, they used a yeast-based directed evolution approach to identify two promising mutations in ADAR2 DD (E620G and Q696L), and incorporated these two mutations, they developed more minor and more precise REPAIR: REPAIR.t1 and REPAIR.t3 systems. The relative efficiency of A-to-I editing achieved nearly 40 ​% on endogenous transcripts in HEK293FT cells [42].

Hui Yang et al. identified and engineered two compact Cas ribonuclease families: type VI-X and VI-Y, which enable single AAV delivery. They fused both dCas13X.1 and truncated dCas13X.1 with ADAR2 DD and generated full-size (xABE) and mini xABE (mxABE), respectively [43]. Later, they used the EcCas6e protein, which is derived from the Class 1CRISPR family, for the construction of ceRBE [44]. These Cas-ADAR RNA editors all have great potential for treating genetic diseases and have achieved up to 60 ​% on endogenous transcripts in HEK293FT cells.

Besides, as compact Cas proteins, the Cas13j family was also utilized for A-to-I editing as mentioned above. The dChi-RESCUE-S, which fuses dChiCas13j with hADAR2 DD, is the smallest A-to-I editor up to 2024 (ChiCas13j is only 424 aa, as the smallest Cas protein) [14].

CIRTS (CRISPR-Cas-Inspired RNA Targeting System) is a modular, programmable RNA effector platform engineered entirely from human protein components. Its core mechanism involves a guide RNA containing a specific RNA hairpin structure recognized by a human hairpin-binding protein, fused to a cationic single-stranded RNA-binding protein for RNA stabilization, and linked to an effector domain – in this case, the catalytic domain of hADAR2 for A-to-I editing. Despite its advantages of small size (enabling AAV delivery), fully human origin (potentially reducing immunogenicity compared to bacterial Cas systems), and modularity for multiplexed targeting, CIRTS faces several challenges. Its editing efficiency and specificity are influenced by the accessibility of the target site on the endogenous RNA and the sequence context requirements of the hADAR2 domain [45].

### 3.2 Endogenous ADARs for A-to-I editing

Over-expression of ADARs is considered necessary for A-to-I editing in earlier studies; most A-to-I editing systems are based on exogenous ADARs. However, some drawbacks limited the therapeutic applications of exogenous ADARs. The first problem is the one mentioned earlier, delivery capacity. Besides, transcriptome-wide off-target effects and immune responses also hinder their development. The over-expression of ADAR1 has been reported to confer oncogenicity in multiple myelomas [46].

Exploiting endogenous ADARs has been unveiled to be reliable for A-to-I editing. Hiroyuki Nakagawa and Thorsten Stafforst et al. independently realized site-specific A-to-I conversion by recruiting ADARs by R/G motif in GluR2 mRNA [47,48]. Since then, using endogenous ADARs has gradually become the mainstream of A-to-I editing.

#### 3.2.1 RESTORE

**Fig. 5:**
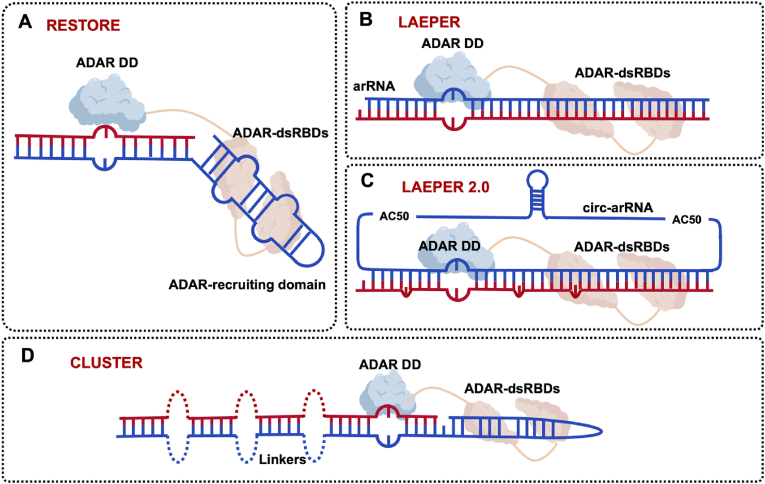
Endogenous ADAR for A-to-I RNA base editing. **A.** Diagram of RESTORE. Chemical modified gRNA with ADAR recruiting domain. **B.** Diagram of LEAPER. Engineered long linear RNA-targeting domain only. **C.** Diagram of LEAPER 2.0. Engineered circular RNA-targeting domain with covalently closed loop. **D.** Diagram of CLUSTER. Add a cluster of single-stranded recruitment sequences on R/G motif gRNA.

Thorsten Stafforst  and Jin Billy Li et al. first used gRNA-alone A-to-I editor, which includes an invariant ADAR recruiting domain (derived from R/G motif in GluR2 mRNA) and a programmable specificity domain to elicit RNA editing, but failed without the over-expression of ADAR2. After that, they turned to test chemically stabilized ASOs instead of plasmid-borne gRNA expression. They exploited a 15-nt chemically modified (2′-O-methylations, phosphorothioate) specificity domain (the editing site locates 9-bp away from the 3’ ends of the gRNA) for A-to-I editing and extended the chemical modification to the ADAR recruiting domain, the later design preformed even better in recruiting endogenous ADAR in HeLa cells, they called this RNA editor RESTORE (recruiting endogenous ADAR to specific transcripts for oligonucleotide-mediated RNA editing) (Fig. 5A). Besides, they treated cells with interferon α (IFNα) to increase the expression of ADAR1 p150 to enhance the A-to-I editing. RESTORE achieved up to 75–85 ​% relative efficiency and high specificity on cell endogenous transcripts [19].

#### 3.2.2 LEAPER and LEAPER 2.0

Wensheng Wei et al. designed arRNA (ADAR-recruiting RNA) to recruit endogenous ADARs and elicit A-to-I editing; they called it LEAPER (leveraging endogenous ADAR for programmable editing of RNA). The arRNA in the LEAPER system is engineered linear and only consists of a long RNA-targeting domain (tens to hundreds of nucleotides, the editing site centrally within the complementary region), and longer arRNA makes higher editing efficiency (Fig. 5B). Unlike the ASOs used in RESTORE systems, arRNA is entirely complementary and symmetrical to the targeted adenosine base and can be produced by chemical synthesis and expression in vivo. LEAPER achieved up to 80% relative efficiency and also high specificity on endogenous transcripts in cells. However bystander off-target editing still existed even though introducing A:G mismatches to increase the editing efficiency [49], mainly due to the formation of long, double-stranded gRNA/mRNA duplexes.

Editing efficiency and durability are vital for RNA base editing, and they depend on the abundance and stability of arRNA. Then they developed LEAPER 2.0 in 2022, which is an updated version of LEAPER, but leverages circular RNA because it is covalently closed loop structure and protects it from exonucleases [50,51] (Fig. 5C). Circularization of arRNA significantly improved the expression level by two to three orders of magnitude, and ∼3.1-fold higher editing efficiency than linear arRNA. The bystander editing events were also ameliorated by deleting the uridines opposite the unintended adenosines in arRNA [28,52].

#### 3.2.3 CLUSTER

Thorsten Stafforst  and Jin Billy Li et al. developed the CLUSTER approach, which is based on their previous R/G-gRNA design (RESTORE). They added a cluster of single-stranded recruitment sequences (RS) on R/G motif. RS bind to the target mRNA in various regions to circumvent bystander bases, distal to the target site and distal to each other (Fig. 5D). The cooperation between RS and a 20-nt programmable specificity domain built a fast and strong binding of guide and target RNA while still keeping the highly flexibility of gRNA properties (the editing site locates 12-bp away from the 3′ ends of the gRNA). The CLUSTER obtained up to 45% editing efficiency and notably reduced bystander editing effects on endogenous transcripts in cells [53].

In 2024, they discovered G•U wobble base pairs in 5′-UAN triplets can effectively mitigate off-target events while maintaining high on-target efficiency. They applied it in a circularized format of the CLUSTER approach, and achieved up to 87% editing efficiency of a disease-relevant mutation in the *Mecp2* transcript in cell culture while further reducing the off-target editing. Besides, introducing of G•U wobble base pairs also improved the precision and efficiency of other endogenous A-to-I editors such as LEAPER [54].

Except for the A-to-I editors mentioned above, many other kinds exist with their own characteristics. MCP-ADAR [55], λN-ADAR [16], *GluR2*-ADAR [17], and CIRTS [45] are based on the interaction between protein and special RNA secondary structures. AI-REWIRE [20] leverages the PUF domain for targeting RNA; these A-to-I editors correct point mutations with exogenous ADAR. The cadRNA [56] based A-to-I editor and AIMers [57] use circular gRNA and chemically modified oligonucleotides for editing with endogenous ADAR, respectively, and they achieve high editing efficiency.

## 4 C-to-U base editors: from evolved ADAR to APOBEC

Unlike the rapid development of A-to-I editors, C-to-U editors are far less common. Even if C-to-U editing is still in the preliminary stage of development, it provides another base conversion choice for RNA editing and therapy.

### 4.1 Evolved ADAR2 DD for C-to-U editing

**Fig. 6:**
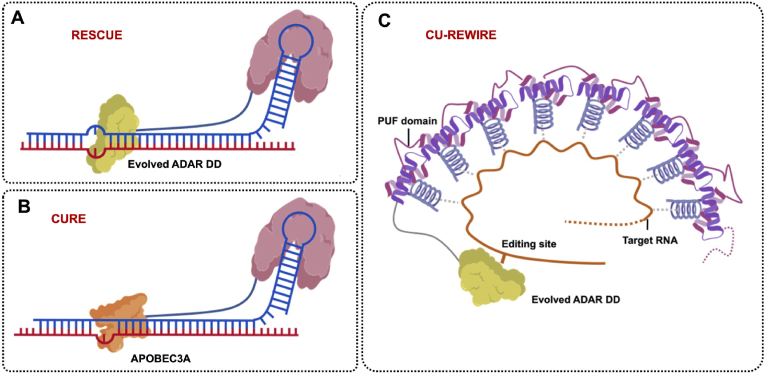
C-to-U editors for RNA base editing. **A.** Diagram of RESCUE. An evolved ADAR DD tethered to a dCas13b. **B.** Diagram of CURE. An APOBEC3A protein tethered to a dCasRx. **C.** Diagram of CU-REWIRE. An evolved ADAR DD tethered to a 10-repeats PUF-domain.

Native cytosine deaminases, which allow the C-to-U conversion, exhibit massive global off-target and bystander off-target when used in DNA base editors. Feng Zhang et al. developed the first RNA C-to-U editor in 2019. They leveraged a synthetic approach to evolve ADAR2 DD, given the high reactivity and specificity of A-to-I editing by ADAR. They constructed cytosine deaminase after 16 rounds of evolution, and 16 mutations are distributed throughout the structure of ADAR2 DD. Then they fused the evolved cytidine deaminase to dCas13 and developed RESCUE (RNA Editing for Specific C-to-U Exchange), and observed editing efficiency between 5 and 28% on β-catenin transcript (*CTNNB1*) (Fig. 6A). Notably, the C-to-U editing by RESCUE was also accompanied by A-to-I editing, because evolved ADAR2 DD still retains adenosine deaminase activity [58]. When they identified Cas13bt1 and Cas13bt3 as mentioned above, they also used these compact Cas proteins in the RESCUE system: RESCUE.t1 and RESCUE.t3. However, this optimization only alleviated the delivery problem but did not significantly improve editing efficiency and off-target issues [42].

Hui Yang et al. also exploited their newly identified two compact Cas ribonuclease families (type VI-X and VI-Y, as mentioned above) for C-to-U editing; this approach achieved up to 70% relative editing efficiency on endogenous transcripts [43].

Except for Cas targeting systems, Thorsten Stafforst et al. developed SNAP-CDAR, which leverages SNAP-tag for targeted nucleotide, just like SNAP-CDAR-S. Compared with the RESCUE systems, the SNAP-CDAR-S performed higher editing efficiency (up to 50%) with less bystander editing, because the chemical design and the covalent bond between guide RNA and SNAP-tag may facilitate the encounter of guide RNA, target RNA, and effector proteins [59].

### 4.2 Fused APOBEC3A for C-to-U editing

APOBEC (apolipoprotein B mRNA editing catalytic polypeptide-like) family mainly contains three members: APOBEC1, APOBEC3A, and APOBEC3G, which introduce the C-to-U mutations [60] (Fig. 3). The C-to-U editing, mediated by APOBEC, is spatially specific rather than widespread, mainly in the small intestine, macrophages, and monocytes [61,62]. With the existence of RNA-binding motif protein 47 (RBM47) [63] and APOBEC1 complementation factor (A1CF) [64], C-to-U editing events act on the mooring sequence, which is a cis-acting RNA structure [65,66]. Due to the similarity between A-to-I editing and C-to-U editing, the targeting systems that C-to-U editing can use are also consistent with the former, no further elaboration is needed here.

Inevitably, C-to-U editing by evolved ADAR2 DD has always accompanied A-to-I off-target editing until now. Tian Chi et al. used APOBEC3A for C-to-U editing, they called it CURE (cytidine-specific C-to-U RNA editor), to mitigate the transcriptome-wide C-to-U editing, they embedded APOBEC3A in the flexible region of dCasRx [67] (Fig. 6B). Zefeng Wang et al. exploited PUF domain and APOBEC3A to construct the REWIRE (RNA editing with individual RNA-binding enzyme) system [20] (Fig. 6C). Over all, these two approaches still exist numerous global off-target C-to-U editing induced by non-specificity deamination of APOBEC3A, and the editing efficiency is no more than 50 ​%, efficient C-to-U editors with low off-target effects still need to be further explored.

## 5 U-to-Ψ RNA base editors: suppressing the nonsense mutations at uracil in PTCs

Pseudouridine (Ψ), one of the most prevalent RNA modifications, is ubiquitously distributed in rRNA, tRNA, snRNA, and mRNA, and functions as the fifth nucleotide in RNA epitranscriptomic regulation. Pseudouridine synthases catalyze this modification through post-transcriptional mechanisms. Its biosynthesis is classified into two distinct pathways: (1) RNA-dependent pathway: Guided by H/ACA box snoRNAs (small nucleolar RNAs), which direct site-specific pseudouridylation in rRNA and other non-coding RNAs. (2) RNA-independent pathway: Enzymes directly recognize structural motifs, such as the TΨC loop in tRNA, to mediate pseudouridine formation without guide RNA. Accumulating evidence has demonstrated that pseudouridine is evolutionarily conserved across species and is critically implicated in diverse pathologies, including cancer, mitochondrial disorders, and immune-related processes. Its dual roles in maintaining RNA structural integrity and modulating immune responses highlight its broad functional significance in cellular homeostasis and disease mechanisms [68,69].

DKC1 (dyskerin pseudouridine synthase 1) is a pseudouridine synthase that enables U-to-Ψ mutations (Fig. 3). Two protein-coding DKC1 isoforms were identified in human cells: DKC1-isoform1 and DKC1-isoform3. Originating from the alternative splicing of DKC1 transcripts, both represent catalytic activity. DKC1-isoform1 is the primary form of DKC1 protein, contains bipartite N- and C-terminal nuclear localization signals (NLSs), and is mainly located in the nucleolus, while DKC1-isoform3 is the minor form, but lacks the C-terminal NLS, which makes it also distributed in the cytoplasm [21,70,71]. The currently developed U-to-Ψ editors are all based on the RNA-dependent pathway (H/ACA box snoRNAs guided pseudouridylation), so the snoRNA/snoRNP systems are directly used as the targeted system.

Single-nucleotide point mutations cause many diseases, of which approximately 20% are nonsense mutations [72]. A nonsense mutation is a type of point mutation in DNA that introduces a premature termination codon (PTC) within the coding sequence of a gene. This occurs when a single nucleotide substitution converts a codon encoding an amino acid into one of the three stop codons (UAA, UAG, or UGA in mRNA) [73]. PTC-containing mRNAs are typically targeted for degradation via the nonsense-mediated mRNA decay (NMD) pathway, a cellular surveillance mechanism to eliminate faulty transcripts [74]. Yitao Yu et al. first demonstrated that RNA-guided RNA pseudouridylation (U-to-Ψ editing) on PTC enables suppression of NMD while promoting PTC readthrough to produce full-length functional protein in the yeast cells, which means pseudouridylated PTCs are no longer recognized as stop codons [75].

**Fig. 7:**
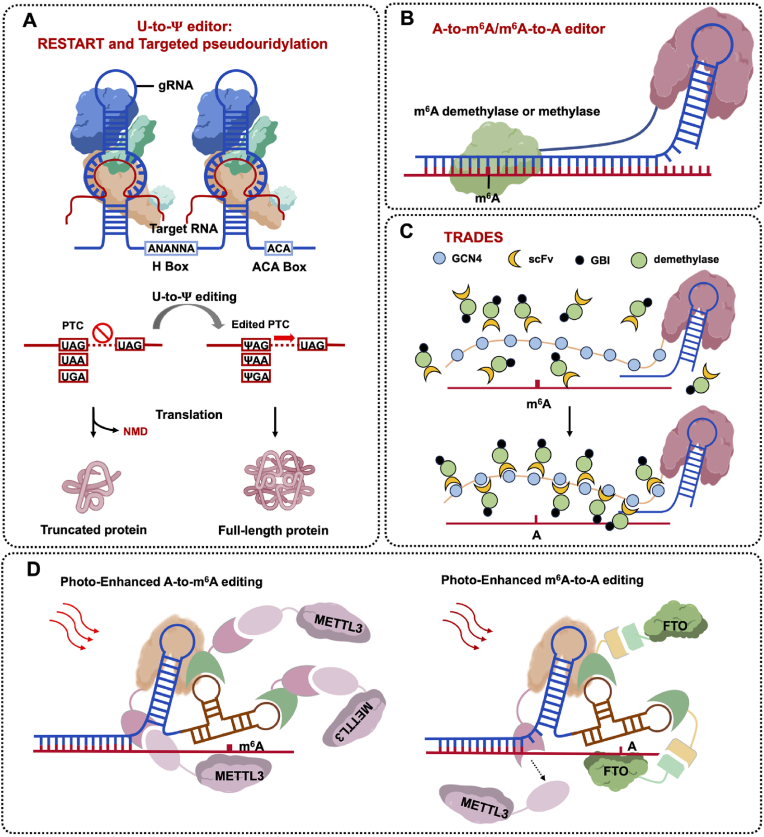
A-to-m^6^A/m^6^A-to-A base editors and U-to-Ψ editors. **A.** Diagram of A-to-m^6^A/m^6^A-to-A base editors. A m^6^A demethylase or adenosine methylase tethered to a dCas protein. **B.** Diagram of TRADES. A dCas13b tethered to SunTag system, and 10 copies of GCN4 peptide enable recruited the scFv-fusion m^6^A demethylase. **C.** Diagram of enhanced or reversible m^6^A editing by red/far-red light induction. Red light triggered ΔphyA-dCas13 recruitment of FHY1-fused METTL3 and A-to-m^6^A editing, Far-red light induced Bphp1-FTO recruitment via PspR2-MCP and m^6^A-to-A editing. **D.** Diagram of RESTART and Targeted pseudouridylation. They used engineered or over-expressed H/ACA box snoRNA for the site-specific pseudouridylation on PTCs to suppress the nonsense mutations.

Chenqi Yi et al. developed RESTART (RNA editing to specific transcripts for pseudouridine-mediated PTC readthrough), which leverages the H/ACA box snoRNP machinery for U-to-Ψ editing. The guide sequences of H/ACA box snoRNAs are approximately a dozen bases long and include in its two hairpins, which contains the H and ACA box motifs, and the programable gsnoRNAs (guide snoRNAs) targeted to a U in PTC site are produced by redesigning the both hairpins (the editing site centrally within the complementary region and is located at the opening of two hairpins). They screened human gsnoRNA scaffolds with high expression levels and engineered them, and this served as the RESTARTv1, which enables PTC readthrough in multiple cell lines (Fig. 7A). An unexpected finding was that the relative editing efficiency of DKC1-isoform3 overexpressed cells were obviously increased; they generated RESTARTv2 with the combination of DKC1-isoform3. RESTART realized site-specific pseudouridylation of PTC in reporters, endogenous transcripts in multiple cell lines and human primary cells, and disease contexts successfully, while the off-target editing events had almost no effect [21]. After that, in 2024, they developed RESTARTv3, which increased ∼5-fold editing efficiency compared to RESTARTv2 by using near-cognate tRNAs. Notably, RESTARTv3 achieved great success in PTC readthrough and protein recovery in cystic fibrosis and Hurler syndrome disease cell models [76].

Yitao Yu et al. also realized site-specific pseudouridylation of PTC in mammalian cells. Unlike the RESTART used hU6-derived H/ACA box snoRNAs, they inserted gRNA (pugU2-34/44) gene into the first intron of the β-globin gene, and they called this approach targeted pseudouridylation. Targeted pseudouridylation also realized long-lasting U-to-Ψ editing, nonsense suppression, and protein recovery in human cells (CFTR and IDUA disease cell models) (Fig. 7A). Besides, they also proved that artificial gRNA intron is a valid approach to lead to nonsense suppression [77].

Similar to the A-to-m^6^A/m^6^A-to-A base editors, U-to-Ψ RNA base editors do not change the sequence either, but they change the genetic code by realizing the PTC readthrough. U-to-Ψ RNA base editing also provides a promising RNA-editing tool for epi-transcriptomic research of the functional roles of individual Ψ modifications, and coping strategies for point mutation disease.

## 6 A-to-m^6^A and m^6^A-to-A base editors: targeted methylation and demethylation on RNA

*N*^6^-methyladenine (m^6^A) is the most common internal modification in eukaryotic mRNA, and it is mainly catalyzed by the methyltransferase complex METTL3-METTL14n(writer), which can be demethylated by FTO or ALKBH5 (eraser), and thus is a reversible chemical modification. As an important epigenetic modification, m^6^A plays an important role in mRNA splicing, polyadenylation, export, translation, stability, and structure [78]. However, the relationships between the specific m^6^A site and phenotypic outcomes were hampered due to the inability to install m^6^A site specifically because of the lack of m^6^A editing tools. The development of the m^6^A editing tools is not only conducive to in-depth understanding of its biological functions, but also expected to become an important tool for regulating cell, thereby providing potential methods for therapeutic.

METTL3-METTL14 (methyltransferase-like 3 and methyltransferase-like 14) complex mediates the methylation of mRNA to form m^6^A (A-to-m^6^A) in mammals (Fig. 3). In this 184kDa heterodimer, METTL3 catalyzes the transfer of a methyl group from S-adenosyl methionine (SAM) to adenine within the single stranded RNA sequence motif Pu(G>A)m^6^AC(A/C/U) (where Pu represents purine, and only a portion of these putative methylation sites contain m^6^A), whereas METTL14 scaffolds substrate RNA binding [23,79].

ALKBH5 (AlkB Homolog 5) is a mammalian RNA demethylase, which belongs to the AlkB family of nonheme Fe(II)/a-ketoglutarate (a-KG)-dependent dioxygenases, which catalyze a wide range of biological oxidations and the removal of the m^6^A modification (m^6^A-to-A) on nuclear RNA in vitro and in vivo [80] (Fig. 3). Though there exist many m^6^A demethylases, ALKBH5 is mostly used in m^6^A-to-A editing.

Currently developed A-to-m^6^A and m^6^A-to-A base editors utilize CRISPR-based RNA-targeting systems. By fusing the effector protein to the Cas protein, these RNA editors achieve site-specific RNA editing by efficient binding of sgRNA to the Cas protein.

Shu-Bing Qian et al. first developed the A-to-m^6^A and m^6^A-to-A base editors in 2019, they fused dCas9 to METTL3-METTL14 and used protospacer adjacent motif (PAM) for targeting, programmable m^6^A modification (A-to-m^6^A) in specific RNA regions (5′ UTR and 3′ UTR) was achieved, and the fusion expression of dCas9 with FTO or ALKBH5 protein can precisely erase the m^6^A modification (m^6^A-to-A) (Fig. 7B). They also demonstrated that a single site 5′ UTR methylation can lead to non-canonical mRNA translation, while manipulating 3′ UTR methylation influences mRNA turnover [22]. David R. Liu et al. tethered dCas13 to m^6^A writers to realize the site-specific programmable m^6^A editing, and they focused on the relationships between specific m^6^A sites and phenotypic outcomes. By fusing the modified methyltransferase components to dCas13, the developed TRM (targeted RNA methylation) system enables site-directed m^6^A installation on endogenous target transcripts in both the cytoplasm and nucleus [23].

Our research group developed TRADES (targeted RNA demethylation RNA editing tool by SunTag system) for m^6^A demethylation. SunTag is a signal amplification system which contains 10 copies of GCN4 peptide enables efficiently recruit the scFv-fusion effector proteins [81,82] (Fig. 7C). By the combination of SunTag and dCas13b, TRADES system can recruit multiple scFv-fusion RNA demethylase to demethylate RNA transcript at specific loci, shows wider editing window than direct dCas9-effector protein conjugates, and more flexible and potential to target the indistinguishable m^6^A sites, while the influence of the off-target editing is negligible. One of the significant advantages of TRADES is that the excess GCN4 peptides can eliminate the unrecruited demethylase, which may cause less perturbation to other endogenous RNA transcripts than forced expression of effector protein alone [83].

Hongsheng Wang et al. developed dm^6^ACRISPR, a fusion protein by linking a dPspCas13b to m^6^A demethylase ALKBH5. This m^6^A-to-A editing tool enables specifically demethylating m^6^A of targeted mRNA with limited off-target events. This programmable tool makes it possible to study specific m^6^A sites of specific genes and their related functions [84]. In the same way, Nan Cao et al. presented the TRME (targeted RNA m^6^A erasure) system to realize m^6^A-to-A editing of RNAs in human embryonic stem cells (hESCs) [85].

Recently, our research group presented an optogenetically controlled CRISPR-dCas13 system for precise and reversible m^6^A editing, enabled by red (660 ​nm) and far-red (740 ​nm) light-inducible heterodimerizing protein pairs: ΔphyA/FHY1 and Bphp1/PspR2. This system integrates engineered dCas13 and sgRNA scaffolds to achieve spatiotemporal modulation of m^6^A modifications. Red light triggered ΔphyA-dCas13 recruitment of FHY1-fused METTL3 (m^6^A methyltransferase), significantly elevating m^6^A levels at specific sites (e.g., 3-fold increase at ACTB A1216) with minimal off-target effects, while Far-red light induced Bphp1-FTO (m^6^A demethylase) recruitment via PspR2-MCP, which enables cyclic m^6^A deposition/erasure at single loci over multiple light cycles (Fig. 7D). Its modular design permits adaptation to other RNA modifications, presenting a versatile tool for probing dynamic epi-transcriptome mechanisms and manipulating cell fate in research and therapeutics [86].

Unlike other RNA base editors, A-to-m^6^A/m^6^A-to-A base editors do not change the genetic code directly. However, just process base editing in the field of epi-transcriptome, offering reversible base editing tools to realize the complicated regulation of transcriptome. A-to-m^6^A/m^6^A-to-A base editors enable elaborate and precise editing of the m^6^A locus to reveal the molecular mechanism and pathological significance of its dynamic modification, have great therapeutic potential and safety advantages.

## 7 Delivery platforms for RNA editing: advancing AAV and LNP technologies

The emergence of programmable RNA editing systems—including SNAP-ADAR, Cas-ADAR, LEAPER, CLUSTER, and RESTART—represents a transformative approach for correcting disease-causing mutations at the transcript level. The research on the vast majority of RNA editors is still at a very basic stage; transfection/co-transfection of plasmids into in vitro cell lines is currently the most commonly used criterion for evaluating the reactivity and targeting efficiency of RNA editors. Though most studies related to RNA editors have not yet been studied in animal models and clinical settings, delivery is undoubtedly a crucial final step for the clinical application of RNA editors. The clinical translation of RNA editors critically depends on delivery vehicles capable of achieving efficient, tissue-specific, and durable in vivo expression. Among current platforms, adeno-associated virus (AAV) vectors and lipid nanoparticles (LNPs) have emerged as leading candidates, offering distinct advantages and limitations.

### 7.1 AAV vectors: mechanisms and therapeutic applications

AAV vectors function as DNA delivery vehicles, transporting expression cassettes encoding RNA editing components (e.g., plasmids which contain the sequence of ADAR2DD and Cas13 nuclease) to target cells. The rAAVs (recombinant AAVs) are composed of the identical capsid sequences and structures as found in wild-type AAVs (wtAAVs). In contrast, rAAVs encapsidate genomes that are devoid of all AAV protein-coding sequences and have therapeutic gene expression cassettes designed in their place [87]. ITRs are the only viral origin sequences needed to guide genome replication and packaging during vector production. By obliterating the viral coding sequencing, the packaging capacity is maximized, and immunogenicity and cytotoxicity are lowered. Following cellular entry via receptor-mediated endocytosis, single-stranded vector genomes convert to transcriptionally active double-stranded DNA episomes. Subsequent host-cell machinery transcribes these cassettes into mRNA, translating them into functional editing proteins that successfully correct pathogenic RNA sequences.

The principal strength of AAV lies in its capacity for long-term expression (several months), and this durability stems from the stability of non-integrated episomes in post-mitotic tissues. Besides, AAV demonstrates relatively low immunogenicity compared to adenoviral or lentiviral vectors, reducing the risk of acute inflammatory responses. Despite these benefits, AAV faces significant constraints: the strict payload capacity (≤4.7 ​kb) impedes delivery of large fusion constructs like SNAP-ADAR, necessitating split-vector approaches that compromise efficiency. While integration events are rare, genomic insertion risks remain a safety concern, particularly with high-dose administration[[88], [89], [90]].

Notable applications include the RESTART v3 platform, which enables it to be packaged into a single viral vector, which is supported by the practical functional rescues in the disease cell models of CF and Hurler syndrome [76].

### 7.2 LNP delivery: engineering innovations and biological performance

LNPs are synthetic nanocontainers (typically 70–100 ​nm) that encapsulate mRNA or ribonucleoprotein (RNP) payloads encoding RNA editors. These nanoparticles enter cells via endocytosis and are composed of ionizable lipids, phospholipids, cholesterol, and PEG-lipids, these nanoparticles enter cells via endocytosis. The ionizable lipids undergo protonation within acidic endosomes, disrupting endosomal membranes to release editing machinery directly into the cytosol. This enables rapid but transient expression—ideal for minimizing off-target effects associated with prolonged editor activity[[91], [92], [93]].

The transient expression profile of LNP-delivered editors significantly reduces the risk of chronic off-target editing (several days). LNPs offer exceptional payload flexibility, accommodating large fusion proteins like SNAP-ADAR, MCP-ADAR, and λN-ADAR without efficiency loss. Furthermore, their synthetic nature permits repeat administration, a critical feature for chronic disorders requiring iterative correction. However, the natural hepatotropic bias of conventional LNPs limits extrahepatic applications; this problem is gradually being solved with the improvement of LNP synthesis methods. Additionally, the inherently short duration of mRNA expression necessitates frequent dosing for sustained efficacy [94].

In addition to the aforementioned delivery containing SNAP-ADAR fusion proteins for RNA editing, most other RNA editors (such as LEAPER) have also researched LNP delivery. Zhang et al. synthesized LNPs that use the FDA-approved ionizable cationic lipid DLin-MC3-DMA to deliver LEAPER circ-arRNA for A-to-I editing. By optimizing the N/P ratio for high transfection efficiency of circ-arRNA and following intratumoral injection of Circ-arRNA LNPs, they observed high and sustained levels of circ-arRNA in vivo and efficient correction of nonsense mutations [95].

AAV and LNP platforms constitute complementary pillars in the therapeutic delivery of RNA editors. AAV excels in achieving durable, tissue-specific expression, particularly valuable for monogenic disorders requiring lifelong correction. Conversely, LNP technology offers superior safety profiles and flexible payload capacity, making it ideal for transient editing applications. The strategic integration of both systems through hybrid approaches mitigates individual limitations while enhancing precision. As emerging platforms like SEND [96,97] and engineered exosomes mature [98,99], they hold the potential to overcome persistent barriers in extrahepatic targeting and immunogenicity. Continued innovation in delivery technologies will undoubtedly accelerate the clinical realization of RNA editing as a transformative therapeutic modality for genetic diseases, cancer, and regenerative medicine.

## 8 Therapeutic applications of RNA editors

Point mutations are alterations in the DNA sequence where a single nucleotide base is substituted for another, inserted, or deleted, representing the smallest scale of genetic change [72]. However, such a small genetic change may cause severe lesions as mentioned in the introduction part. Nonsense mutations, characterized by premature termination codon (PTC) introduction via single-base substitutions, account for >20% of disease-causing point mutations and lead to truncated nonfunctional proteins or mRNA degradation through nonsense-mediated decay (NMD) [73]. In recent years, RNA editing technologies have emerged as promising therapeutic strategies to correct PTCs at the transcript level, circumventing genomic permanence risks while restoring functional protein expression.

### 8.1 RNA editing for Hurler syndrome

Hurler syndrome, the most severe form of mucopolysaccharidosis type I, arises from autosomal recessive mutations in the *IDUA* gene, with nonsense mutations (e.g., W392X) accounting for >40% of pathogenic alleles. These mutations introduce premature termination codons (PTCs) into *IDUA* mRNA, leading to truncated, non-functional α-_L_-iduronidase enzyme. Consequently, glycosaminoglycans (GAGs) accumulate in lysosomes, driving progressive multisystem deterioration—including neurodegeneration, skeletal dysplasia, and organ failure [100,101]. Current therapies, including allogeneic hematopoietic stem cell transplantation (HSCT) [102] and enzyme replacement therapy (ERT), remain suboptimal due to reasons such as high treatment costs, lifelong medication, and potential immune responses. The development of RNA base editors will provide new treatment options.

Wei et al. designed two versions of LEAPER arRNA with chemical modifications, targeting the PTC (W392X) of mature mRNA and the pre-mRNA of IDUA in primary fibroblast GM06214 ​cells, that was originally isolated from a Hurler syndrome patient, respectively. Both arRNA edited the termination codon (UAG) to the codon encoding tryptophan (UGG), significantly restored the IDUA catalytic activity, and neither induced expressions of genes involved in type-I interferon and pro-inflammatory responses [49]. They also designed two versions of LEAPER 2.0 circ-arRNA and delivered them to Idua-W392X mice via transduction of a self-complementary AAV. Both achieved a ∼10% targeted editing rate and significantly restored IDUA catalytic activity [52]. These results all demonstrate that LEAPER and LEAPER 2.0 can serve as precise and efficient treatment tools for gene mutation diseases.

In addition, the U-to-Ψ base editors were also used to suppress the nonsense mutation of IDUA-W392X in mice. Yi et al. delivered RESTARTv2 into primary mouse embryonic fibroblasts and observed ∼12.1 ​% mutation rate on endogenous transcript [21]. Yu et al. also applied their target pseudouridylation approach to the Hurler syndrome cell model and achieved ∼7%–9% rescue of full-length IDUA protein [77].

### 8.2 RNA editing for cystic fibrosis

Cystic fibrosis (CF) arises primarily from mutations in the *CFTR* gene, nearly 10% are nonsense mutations, which introduce premature termination codons (PTCs) within the *CFTR* mRNA, leading to truncated, non-functional CFTR protein and a complete loss of chloride channel activity at the epithelial surface. Elexacaftor/tezacaftor/ivacaftor (ETI), a triple-combination modulator approved in 2019 that benefits ∼90 ​% of patients carrying at least one F508del allele, but has little effect on the remaining 10 ​% of patients who are caused by nonsense mutations [103]. RNA-targeted interventions, particularly those employing precise editing technologies, offer a promising avenue to directly correct the PTC or restore functional protein expression, potentially addressing the root cause in this refractory patient subset.

The strategy of suppressing the nonsense mutation by U-to-Ψ base editors also makes sense in CF cell models. Yi et al. delivered RESTARTv2 into HEK293T cells that were transfected with CFTR-R553X or CFTR-W1282X expressing plasmids. They observed that the function of CFTR was rescued to about 30%–40% of the CFTR-WT level in two cell models and a 37.2% readthrough level of CFTR-R553X compared with CFTR-WT [21]. Yu et al. transfected target pseudouridylation designer gRNA into 16HBEge-G542X (16HBE14o-bronchial epithelial cells), which carries a PTC (G542X) in the endogenous (chromosomal) CFTR gene. They realized ∼9%–12% and ∼6 ​%–8% restoration of mRNA and full-length protein, respectively [77].

Beyond the aforementioned two diseases, disorders caused by genetic mutations—such as peroxisome biogenesis disorders (PBDs) [21], Duchenne muscular dystrophy (DMD) [104,105], Rett syndrome [106], and mutations in the *TP53* tumor suppressor gene [49]—are also potential candidates for treatment with A-to-I and U-to-Ψ editing. The therapeutic approach of RNA editing has been preliminarily applied to cellular models of these diseases. Although substantial challenges remain before the clinical application of RNA editing can be realized, this strategy offers a promising alternative therapeutic avenue for managing these conditions.

## 9 Conclusions: challenges and prospects of RNA base editing

RNA base editing technology facilitates the precise modification of specific nucleotides within RNA molecules, providing a transformative tool for functional genomics, therapeutic development, and synthetic biology. Despite its immense potential for advancement, this field encounters multifaceted challenges. Below, we analyze the critical challenges and opportunities in current research.

Existing RNA editors developed to date cannot achieve all possible base-to-base conversions or transformations between canonical bases and their modified counterparts. Notably, A-to-I editors represent the most advanced single-base editing systems, having achieved significant breakthroughs in editing efficiency and mitigating off-target effects. However, C-to-U editing still exhibits substantial room for improvement in reaction efficiency and off-target activity. In contrast, A-to-m^6^A and U-to-Ψ editors, which do not alter the RNA sequence, demonstrate markedly lower off-target side effects than the former systems, though their editing efficiencies remain suboptimal. Current RNA editor delivery efforts focus on engineering RNA editors with enhanced compatibility to existing delivery modalities. A key strategy involves the development of compact Cas protein variants (e.g., miniature Cas13 orthologs) to minimize steric constraints during encapsulation and cellular uptake. Synergistic optimization of delivery systems is imperative, including incorporating tissue-specific targeting ligands into lipid nanoparticles (LNPs) or adeno-associated virus vectors to augment organ- and cell-type selectivity. Furthermore, while the transient nature and dose-dependent controllability of RNA editing represent its most prominent advantages, critical questions remain to be addressed: whether clinical applications for chronic diseases will necessitate chronic therapeutic administration, and whether persistent RNA editing activity may incur unforeseen risks. These concerns demand rigorous preclinical evaluation and longitudinal safety validation.

In conclusion, RNA editing is poised to emerge as a transformative therapeutic modality, leveraging its unique capability for dynamic and reversible modulation of cellular processes. Future advancements will focus on multidimensional innovation: (1) Computational design integration. Deployment of deep learning architectures to predict RNA secondary structures and optimize gRNA targeting specificity. (2) Enzyme engineering breakthroughs. Structure-guided evolution of effector proteins and fusion proteins with enhanced RNA recognition flexibility. (3) Spatiotemporal control systems. Development of chemically inducible editors enabling dose-titratable editing with subcellular precision. (4) Epi-transcriptomic network integration. Systems-level analyses elucidating crosstalk between RNA editing and dynamic modifications.

Advancements in developing of diversified RNA editors are set to revolutionize their utility in fundamental research and clinical therapeutics. The progressive refinement of catalytic efficiency and targeting specificity may position RNA editing as a competitive alternative to CRISPR-Cas9 genome editing.

## CRediT authorship contribution statement

**Weikai Yan:** Writing – original draft, Resources. **Xiaocheng Weng:** Writing – review & editing, Supervision.

## Ethics approval

Not applicable.

## Declaration of generative AI in scientific writing

No generative AI tools have been used throughout the entire writing process of this manuscript.

## Funding information

This work was supported financially by the Noncommunicable Chronic Diseases-10.13039/501100018537National Science and Technology Major Project (2023ZD0507700,2023ZD0507701), the 10.13039/501100001809National Natural Science Foundation of China [92253202 and 22177087 to X. W.].

## Data availability

Not applicable.

## Declaration of competing interest

The authors declare that they have no known competing financial interests or personal relationships that could have appeared to influence the work reported in this paper.
